# Pharmacokinetics, Tissue Residues, and Withdrawal Times of Oxytetracycline in Rainbow Trout (*Oncorhynchus mykiss*) after Single- and Multiple-Dose Oral Administration

**DOI:** 10.3390/ani13243845

**Published:** 2023-12-14

**Authors:** Orhan Corum, Duygu Durna Corum, Ertugrul Terzi, Kamil Uney

**Affiliations:** 1 Department of Pharmacology and Toxicology, Faculty of Veterinary Medicine, University of Hatay Mustafa Kemal, Hatay 31060, Türkiye; ddurnacorum@gmail.com; 2 Department of Veterinary Medicine, Devrekani TOBB Vocational School, University of Kastamonu, Kastamonu 37200, Türkiye; ertugrulterzi@gmail.com; 3 Department of Pharmacology and Toxicology, Faculty of Veterinary Medicine, University of Selcuk, Konya 42031, Türkiye; kuney@selcuk.edu.tr

**Keywords:** antibiotics, aquaculture, drug residue, food safety, public health

## Abstract

**Simple Summary:**

Determination of the pharmacokinetics of oxytetracycline (OTC) at single and multiple oral doses revealed its long half-life, very low bioavailability, and strong accumulation in rainbow trout. The withdrawal time (WT) for the safe consumption of rainbow trout muscle+skin varied according to the guidelines set by regulatory authorities in different countries. This study improves the establishment of the optimal dosing regimen following OTC multiple administration and the determination of the appropriate WT for the safety of rainbow trout consumption.

**Abstract:**

The aim of this study was to compare the pharmacokinetics of oxytetracycline (OTC) following single- (60 mg/kg) and multiple-dose oral administrations (60 mg/kg, every 24 h for 7 days) in rainbow trout. It also aimed to determine bioavailability after a single dose and tissue residues and withdrawal times after multiple doses. This study was carried out on 420 rainbow trout at 9 ± 0.8 °C. This study was carried out in two stages: single-dose (intravascular and oral) and multiple-dose treatment. The OTC concentrations in plasma and tissues were measured by high-performance liquid chromatography and analyzed by a non-compartmental method. The withdrawal time (WT) was estimated using the WT 1.4 software. OTC exhibited a long terminal elimination half-life (t_1/2ʎz_) after IV and oral administration. The oral bioavailability of OTC was very low (2.80%). In multiple-dose treatment, t_1/2ʎz_, the area under the plasma concentration–time curve and peak plasma concentration increased significantly after the last day compared to the first day. OTC showed strong accumulation after multiple doses with a value of 5.33. OTC concentrations were obtained in the order liver > kidney > muscle+skin > plasma. At 9 ± 0.8 °C, the WT calculated for muscle+skin was 56 days for Europe and 50 days for China, respectively. The t_1/2ʎz_ (68.94 h) and time (68 h) above the 1 µg/mL MIC following a single OTC dose may support the extension of the 24 h dosing interval following multiple dosing. However, further studies are required to determine the optimal dosage regimen in multiple-dose OTC treatment in the treatment of infections caused by susceptible pathogens.

## 1. Introduction

As people have changed their dietary habits for a healthy diet and increased their fish consumption, farms have started growing fish to meet the increasing need. However, adverse conditions such as stock density, inadequate feeding, and sudden changes in water temperature have caused the spread of bacterial infections in fish, which cause significant economic losses and deaths [1]. Rainbow trout (*Oncorhynchus mykiss*) is one of the world’s most cultivated fish species, accounting for 1.6% (848.1 tons) of the total global production from fisheries in 2018 [2]. Rainbow trout has a very high economic importance in food production due to its features such as rapid growth, tolerance to relatively high temperatures, and suitability for hatchery cultivation [3].

Rainbow trout commonly experiences diseases such as bacterial hemorrhagic septicemia, furunculosis, enteric red mouth disease, and rainbow trout fry syndrome [4]. Antibiotics are commonly used in the treatment of these bacterial diseases in rainbow trout. Antimicrobial residues in food have received considerable attention due to rising food safety and public health concerns. The most significant effects of antimicrobial residues on public health are the development of antimicrobial drug resistance, the disruption of intestinal flora, hypersensitivity reactions, the depression of bone marrow, teratogenicity, carcinogenicity, and mutagenicity [5]. To protect human health, antimicrobial residue levels in foods must be below the maximum residue limit (MRL) values [6]. Therefore, there is a need to determine the pharmacokinetics, tissue residue, and withdrawal time of antimicrobials in fish [7].

Oxytetracycline (OTC) is a tetracycline-group antibiotic widely used in bacterial fish diseases. OTC has been used in fish for many years due to its advantages such as having a wide spectrum of action, good penetration, low cost, and minimal toxic effects [8]. OTC has a bacteriostatic effect and prevents bacterial protein synthesis by binding to the 30S ribosomal subunit of susceptible bacteria and blocking the mRNA binding of tRNA [9]. OTC is the first-choice antibiotic in the treatment of septicemia, furunculosis, enteric red mouth disease, rainbow trout fry syndrome, and vibriosis diseases in fish and is used orally at a dose of 60–100 mg/kg every 24 h for 5–10 days [4,9,10]. The MRL of OTC for muscle+skin in fish was 100 μg/kg in the European Union (EU) and 200 μg/kg in China [11,12,13].

Although many studies have been reported on the oral single-dose pharmacokinetics of OTC in fish, studies on the multiple-dose pharmacokinetics are limited [14]. It has been reported that the elimination half-life and plasma concentration of OTC significantly increased and accumulated in the body after multiple-dose administration in blunt-snout bream [14]. OTC is used in multiple doses for bacterial fish infections [15]. Although there are many studies on single-dose pharmacokinetics and tissue residue after repeated administration of OTC in rainbow trout [16], no studies on multiple-dose pharmacokinetics have been found. OTC exhibits a long elimination half-life due to slow elimination after single-dose administration in rainbow trout [17]. Therefore, it was hypothesized that multiple doses of OTC to rainbow trout may cause the saturation of pharmacokinetic pathways and accumulation in the body, resulting in changes in pharmacokinetics. The aim of the present study was as follows: (a) to determine the pharmacokinetics and bioavailability of OTC after a single intravenous (IV) and oral administration of 60 mg/kg; (b) to investigate the pharmacokinetic behavior and accumulation of OTC after multiple-dose oral administrations, and (c) to establish tissue residues and the withdrawal time of OTC following multiple-dose oral administration.

## 2. Materials and Methods

### 2.1. Chemicals

Oxytetracycline hydrochloride (≥95%) analytical standard was purchased from Tokyo Chemical Industry (Tokyo, Japan) in powder form. High-performance liquid chromatography (HPLC)-grade acetonitrile and other chemicals were purchased from VWR International (Fontenay-sous-Bois, France). OTC commercial preparations were used for IV (Pan-Terramycin, 30 mg/mL, Injection Solution, Zoetis, Istanbul/Turkiye) and oral (Oksifish 75% Medicated Premix, Medicavet, Istanbul/Turkiye) administration to fish. The oral preparation of OTC was administered to fish after dissolving (30 mg/mL) with injection water.

### 2.2. Animals

From a culture pond in Kastamonu city in Turkiye, 420 healthy rainbow trout (mean body weight, 155 ± 15 g) with no history of drug use in the last two months before this study were obtained. Prior to this study, fish were visually examined for signs of disease, poor body condition, and trauma. The experimental procedure on fish was carried out in concrete ponds with a continuous flow of spring water (pH: 8.1 ± 0.2, water temperature: 9 ± 0.8 °C) within the fish farm. Fish were kept under natural photoperiod conditions with their natural daily cycle. The fish were placed in the ponds for two weeks before this study to acclimate to the environment and were fed with drug-free commercial feed (Sibal, Sinop, Turkiye). The fish were starved 24 h before single-dose treatment and on the days of drug administration in multiple-dose treatment.

### 2.3. Experimental Design

In single-dose and multiple-dose groups, drug administration and blood collection were performed under tricaine methanesulfonate (MS-222, 200 mg/L) anesthesia. Each fish was sampled only once to minimize handling and immobilization stress. Six fish from each group were used at each sampling time. Blood samples were collected from caudal vessel using a 26 G needle attached to a 1 mL syringe.

#### 2.3.1. Single-Dose Pharmacokinetic Study

A total of 216 fish were randomly divided into two groups for IV and oral treatment. Additionally, each group was equally divided into 18 subgroups. OTC was administered through IV (n = 108, caudal vessel) and oral (n = 108, via the gastric gavage) routes at 60 mg/kg dose. Blood samples (1 mL) were collected into heparin-containing anticoagulant tubes at 0 (control), 0.25, 0.5, 1, 2, 4, 8, 12, 24, 48, 72, 96, 120, 144, 192, 240, 288, and 360 h post dosing. Blood samples were centrifuged at 4000× *g* for 10 min, and the resulting plasma was kept at 80 °C until analysis.

#### 2.3.2. Multiple-Dose Pharmacokinetic Study

A total of 204 fish were randomly divided into 34 subgroups. OTC was administered to fish orally (via the gastric gavage) at a dose of 60 mg/kg every 24 h for 7 days. Blood samples were collected in heparin-containing anticoagulant tubes at 0 (control), 0.25, 0.5, 1, 2, 4, 8, 12, and 24 h on the first (1 dose) day and at 0.25, 0.5, 1, 2, 4, 8, 12, 24, 48, 72, 96, 120, 144, 192, 240, 360, 480, 600, 720, 840, 960, 1080, 1200, 1440, and 1920 h on the last (7 doses) day. In addition, muscle+skin, kidney, and liver samples were taken after the last dose administration to determine the tissue residue and withdrawal time of OTC. Blood samples were centrifuged at 4000× *g* for 10 min, and the resulting plasma and tissue samples were kept at 80 °C until analysis.

### 2.4. Oxytetracycline Analysis

The determination of OTC in plasma and tissue was carried out using slightly modified, previously published HPLC-UV (Shimadzu, Tokyo, Japan) methods [6,18]. In brief, tissues were homogenized using a homogenizer (Heidolph Silent Crusher M, Schwabach, Germany) at 12,000 rpm for 40 s. Following that, 100 μg tissue and 100 μL plasma were treated with 200 μL of buffer/EDTA (0.1 M disodium EDTA containing 0.1 M sodium phosphate) and 50 μL of perchloric acid (60%). After vortexing for 1 min, the mixture was centrifuged at 15,000× *g* for 12 min. Transferring the clean supernatant to autosampler vials, 50 μL was injected into the HPLC system. HPLC system consists of a UV–VIS detector (SPD-20A), a column oven (CTO-10A), a degasser (DGU-20A), a pump (LC-20AT), and an auto-sampler (SIL 20A). Chromatographic separation of OTC was performed using an inertsil ODS-3 column (4.6 × 250 mm; 5 μm; GL Sciences, Tokyo, Japan) and the wavelength was set at 260 nm. The temperature of the column and auto-sampler were maintained at 40 °C and 24 °C, respectively. The mobile phase, flowing at a rate of 0.8 mL/min, was composed of acetonitrile (20%) and trifluoroacetic acid in water (80%, 0.01 M).

The chromatographic method was validated in accordance with guidelines from the European Medicines Agency [19]. The OTC hydrochloride stock solution was made by dissolving it in purified water to achieve a concentration of 10 mg/mL. Since OTC exhibits a concentration of 0.04–945 μg/mL (g) in plasma and tissue, both working and calibration standards were prepared in two ranges: range 1 (0.04–40 μg/mL (g)) and range 2 (40–1000 μg/mL (g)). OTC exhibited good linearity with a correlation coefficient of >0.9993 following the concentration ranges of 0.04–40 μg/mL (g) and 40–1000 μg/mL (g). To check the recovery, precision, and accuracy of the method, three calibration samples with low (range 1: 0.04 μg/mL (g), and range 2: 40 μg/mL (g)), medium (range 1: 4 μg/mL (g), and range 2: 400 μg/mL (g)), and high (range 1: 40 μg/mL (g), and range 2: 1000 μg/mL (g)) concentrations for each range were analyzed. The recoveries of OTC were >90% in plasma and were >85% in tissues. The intra-day and inter-day bias obtained in the two calibration ranges were ±6.3 and ±7.4%, respectively. The intra-day and inter-day precision with coefficients of variation <6.5% and <7.8%, respectively, had values below the limit accepted (15%).

### 2.5. Pharmacokinetic Analysis

Pharmacokinetic non-compartmental analysis of OTC concentration–time data was analyzed using the WinNonlin 6.1.0.173 software (Pharsight Corporation, Scientific Consulting Inc., Raleigh, NC, USA). Pharmacokinetic parameters of OTC were calculated from the mean plasma concentration values collected at each sampling time, as reported in previous studies [20,21]. The pharmacokinetic parameters calculated included the area under the plasma concentration–time curve (AUC), AUC extrapolated from tlast to ∞ in % of the total AUC (AUC_extrap_ %), volume of distribution at steady state (V_dss_ = Cl_T_ × MRT), apparent volume of distribution (V_darea_), total body clearance (Cl_T_ = Dose/AUC), terminal elimination half-life (t_1/2ʎz_), mean residence time (MRT), peak plasma concentration (C_max_), time to reach C_max_ (T_max_), plasma concentration at time 0.25 h (C_0.25h_), and bioavailability (F = AUC_oral_/AUC_IV_). AUC_IV_ and AUC_oral_ were determined using the linear/log trapezoidal and linear up/log down methods, respectively. The accumulation ratio (R) of the OTC in the plasma was calculated using the following formula [22], R = AUC_(0–24)ss_/AUC_(0–24_)1, where AUC_(0–24)ss_ and AUC_(0–24)1_ are the areas under the curve calculated for the last (day 7) and first (day 1) dose administration, respectively.

### 2.6. Withdrawal Time Analysis

The withdrawal time (WT) of OTC was determined using the EMA-developed WT 1.4 software [23]. WT was calculated via linear regression analysis considering reported MRLs as the cut-off and is expressed in days. WT was calculated for edible tissues only, and the MRL for muscle+skin of OTC in fish was 100 μg/kg in the EU community and 200 μg/kg in China [11,12,13]. Because the WT 1.4 software can only analyze datasets with a maximum of seven time points, only a portion of each dataset was utilized for the WT 1.4 analysis in this study. For the calculation of WT, a tolerance limit based on the 95th percentile with a confidence level of 95% was used [23].

### 2.7. Statistical Analysis

Plasma and tissue concentrations of OTC are presented as mean ± SD. The following formula was used to determine differences in pharmacokinetic parameters: [100 × (Value obtained from Y − Value obtained from X)/Value obtained from X]. The values of ≤%(−) 25 and ≥%(+) 25 values were accepted as significant [24].

## 3. Results

### 3.1. Single-Dose Pharmacokinetic Study

**Figure 1:**
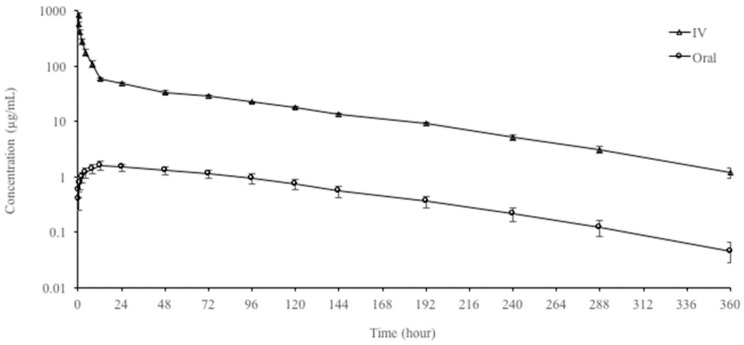
Semi-logarithmic plasma concentration–time curves of oxytetracycline following single intravenous (IV) and oral administrations of 60 mg/kg dose in rainbow trout at 9 ± 0.8 °C (n = 6, mean ± SD).

The semi-logarithmic plasma concentration–time curves and pharmacokinetic parameters of OTC following the single IV and oral administration of 60 mg/kg to rainbow trout are shown in Figure 1 and Table 1, respectively. OTC was detected in plasma up to 360 h after IV and oral administration. OTC following IV and oral administrations exhibited a long t_1/2ʎz_ of 65.05 to 68.94 h, and there was no difference between administration routes. The V_darea_ and Cl_T_ after IV administration were 758.74 mL/kg and 8.09 mL/h/kg, respectively. OTC reached a C_max_ of 1.61 ± 0.25 µg/mL at 12 h after oral administration. The oral bioavailability of OTC was very low with a value of 2.80%.

### 3.2. Multiple-Dose Pharmacokinetic Study

**Figure 2:**
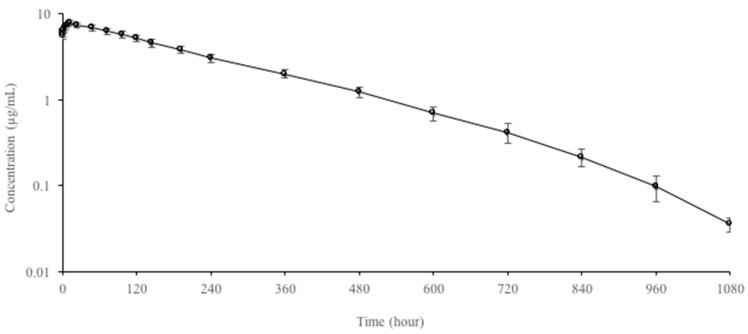
Semi-logarithmic plasma concentration–time curves of oxytetracycline following multiple-dose (every 24 h for 7 days) oral administration of 60 mg/kg in rainbow trout at 9 ± 0.8 °C (n = 6, mean ± SD).

Semi-logarithmic plasma concentration–time curves and pharmacokinetic parameters of OTC following multiple-dose (every 24 h for 7 days) oral administration at a dose of 60 mg/kg to rainbow trout are presented in Figure 2 and Table 2, respectively. It was detected in plasma up to 1080 h after OTC multiple-dose administration. After multiple-dose administration, t_1/2ʎz_, AUC_0–1080_, and C_max_ values of OTC were 147.26 h, 1978.98 h·µg/mL, and 7.82 ± 0.42 µg/mL, respectively. Compared to single-dose administration, t_1/2ʎz_, AUC_0–24_, and C_max_ values increased 2.14, 9.2, and 4.85 times, respectively, after multiple-dose administration. Pharmacokinetic parameters on the first day and the last day after multiple-dose oral administration are presented in Table 3. OTC showed strong accumulation with a drug accumulation level of 5.33 on day 7 after multiple-dose oral administration.

### 3.3. Residue Depletion Study and Withdrawal Time Estimation

Plasma and tissue concentrations of OTC after multiple-dose (every 24 h for 7 days) oral administration to rainbow trout are presented in Table 4. OTC was detected in plasma, liver, kidney, and muscle+skin up to 1080, 1440, 1440, and 1200 h, respectively. OTC concentration levels were obtained in the order liver > kidney > muscle+skin > plasma. The highest OTC concentrations for muscle+skin, liver, kidney, and plasma were 18.23 ± 1.41, 172.66 ± 23.41, 24.40 ± 1.59, and 7.82 ± 0.42 µg/mL, respectively. These results show that OTC has a particular affinity for the liver in rainbow trout.

**Figure 3:**
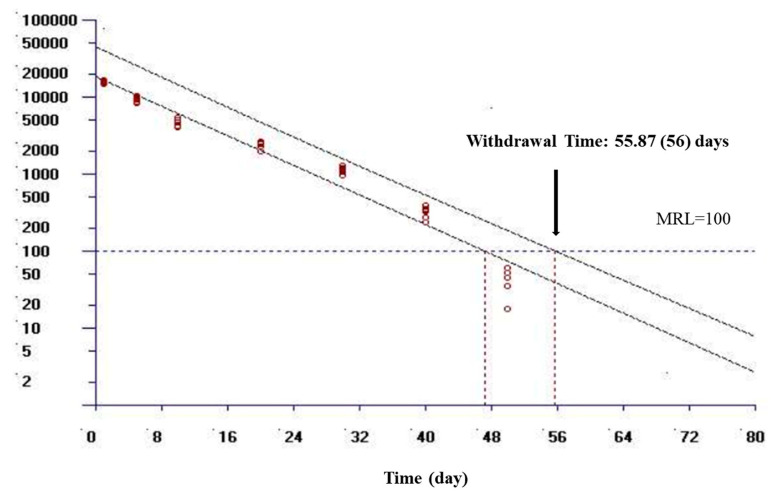
Calculated withdrawal times for muscle+skin of oxytetracycline following multiple-dose (every 24 h for 7 days) oral administration of 60 mg/kg in rainbow trout at 9 ± 0.8 °C. The withdrawal time was calculated by setting the maximum residue limits (MRLs) for Europe using the WT 1.4 software developed by European Medicine Agency [23]. If the calculated withdrawal time was a fraction of a day, the estimated withdrawal time is provided in parentheses, which was rounded up to the next day.

The calculated WT values of OTC for muscle+skin based on MRL values are presented in Table 5. At 9 ± 0.8 °C, the calculated WT for muscle+skin was 56 and 50 days, respectively, considering the MRL values reported by the EU and China (Figure 3).

## 4. Discussion

Oxytetracycline is one of the most common antibiotics used in aquaculture. It is used orally in OTC bacterial fish diseases at a dose of 60–100 mg/kg every 24 h for 5–10 days [25]. Although OTC has been used repeatedly to treat bacterial fish diseases, little is known about how its pharmacokinetics change after repeated administration. In this study, the pharmacokinetics and body accumulation ratio of OTC after multiple-dose oral administration in rainbow trout were investigated for the first time. In fish, drugs are generally applied in the form of medicated feed due to the ease of administration and low labor force [26]. However, we administrated OTC via oral gavage to be able to apply a precise dose to rainbow trout and to prevent possible drug loss due to feed.

The t_1/2ʎz_, Cl_T_, and V_darea_ values following IV administration of OTC in rainbow trout at 9 ± 0.8 °C were 65.05 h, 8.09 mL/h/kg, and 758.74 mL/kg, respectively. The t_1/2ʎz_, Cl_T_, and V_darea_ of OTC following IV administration at different doses (5–60 mg/kg) and different temperatures (10–16 °C) in rainbow trout were reported as 33.51–94.22 h, 6.43–25.40 mL/h/kg, and 870–2990 mL/kg, respectively [17,27,28,29,30,31]. Consistent with previous studies on rainbow trout, OTC exhibited a long t_1/2ʎz_, low Cl_T_, and wide V_darea_ in our study. The most important reason these parameters vary widely in rainbow trout is the temperature difference. Additionally, differences in age/size, pharmaceutical formulation, dosage, health status, and analysis method may have an impact [16].

The C_max_ after the single oral administration of OTC in rainbow trout at a dose of 60 mg/kg at 9 ± 0.8 °C was 1.61 μg/mL. The C_max_ after oral administration of OTC in rainbow trout was 5.77 μg/mL at a dose of 50 mg/kg (gelatin capsule by gastric gavage, temperature: 11 °C, body weight: 350 g) [29] and 2.00–5.30 μg/mL at a dose of 75 mg/kg (aqueous suspension by gastric gavage, temperature: 5–16 °C, body weight: 246–558 g) [17,32]. The difference in C_max_ of OTC in rainbow trout may be due to changes in drug formulations, temperature, and body size. The oral bioavailability of OTC in rainbow trout was very low at 2.80%. The low bioavailability of OTC (0.6–5.6%) has been reported in previous studies on rainbow trout [16,28]. OTC is digested from the gastrointestinal tract by 60% in humans and 7–9% in rainbow trout [33]. OTC is known to form chelates with di- and trivalent ions in feed and water, and this impairs absorption from the intestines [34]. The pH of the duodenum in humans and rainbow trout is 6 and 9, respectively [35]. The fact that the duodenum in fish is more basic may have caused the acidic OTC to be more ionized and therefore the absorption to be lower. This may explain the low bioavailability of OTC in rainbow trout. However, a study conducted on rainbow trout reported that the oral bioavailability of OTC was 30.30%. The reason for the high bioavailability was shown to be the application of OTC by dissolving it in methanol [29].

The multiple-dose drug administration is carried out before the plasma concentration of the previous dose declines below a specific drug concentration and the drug gradually accumulates in the body until it reaches steady state [36]. It is generally assumed that when the drug dosing interval is equal to t_1/2ʎz_, the steady-state concentration can be achieved with 5–6 repeat doses [37]. When many studies in fish are examined, the average t_1/2ʎz_ of OTC is reported to be 83 h, but the standard dosing interval of OTC in fish is 24 h [16]. Compared with single-dose administration, t_1/2ʎz_, AUC_0–∞_, and C_max_ of OTC increased significantly after multiple-dose administration. Similar results have also been observed in the blunt-nosed sea bream [14] and the Pacific white shrimp [15] after a multiple-dose administration. OTC is mainly eliminated unchanged in the urine and bile fluid of mammals [32]. The t_1/2ʎz_ of OTC after single-dose oral administration in rainbow trout was very long at 68.94 h. Therefore, the change in pharmacokinetic parameters after the multiple-dose administration of OTC in rainbow trout at a 24 h dose interval may be due to saturation in the elimination pathways. In this study, the accumulation ratio of OTC was 5.33. The accumulation ratio of OTC in blunt-nosed sea bream is reported as 2.02 [14]. The accumulation ratio of drugs is defined as weak (1.2 ≤ R < 2), moderate (2 ≤ R < 5), and strong (R ≥ 5) [38]. These data show that the oral administration of OTC every 24 h for 7 days causes strong accumulation in the body. Because OTC accumulated significantly in rainbow trout after multiple-dose treatment, reducing the dose, increasing the dosing interval, or both should be considered when creating a dosage regimen.

In rainbow trout, after the daily oral administration of 60 mg/kg for 7 days at 9 ± 0.8 °C, the highest concentrations obtained for the muscle+skin, liver, kidney, and plasma were 18.23 ± 1.41, 172.66 ± 23.41, 24.40 ± 1.59, and 7.82 ± 0.42, respectively. OTC concentration levels were obtained in the order liver > kidney > muscle+skin > plasma. The liver concentration of OTC was 9.43, 7, and 24.5 times higher than muscle+skin, kidney, and plasma concentrations, respectively. The V_darea_ after IV administration was 758.74 mL/kg, which was close to previously reported (870–2988 mL/kg) values in rainbow trout [29,31]. Although OTC is a lipophilic drug, its lipophilicity is lower than those of other tetracyclines [39] and its plasma protein binding ratio varies between 25 and 62% in rainbow trout [16,28]. The reason why the tissue concentration of OTC is high in plasma may be due to its lipophilic structure and partially low binding to plasma proteins. Similar to our research, the maximum concentration was found in the liver of fish in other studies [10,40,41,42]. The kidney in mammals and birds and the liver in fish play important roles in the excretion of OTC [32,43,44]. The reason OTC is found in high concentration in the liver in fish may be that it is the elimination organ.

In this study, the WT 1.4 software program was used to calculate WT values. WT calculation in fish is recommended for muscle+skin, which are edible tissues [23]. The presence of antibiotic residues in edible tissue could lead to the development of bacterial resistance and toxicity to consumers [5]. Therefore, the antibiotic residue in edible tissue should be below the MRL for food safety. The MRL may vary from country to country depending on local food safety regulators and drug use habits [5]. The MRL for muscle+skin is reported as 100 μg/kg and 200 μg/kg in Europe and China, respectively [11,12,13]. At a water temperature of 9 ± 0.8 °C, the calculated WT for muscle+skin was 56 and 50 days, respectively, considering the MRL values reported by the EU and China. The calculated WT in muscle tissue for OTC in rainbow trout was 37–92 days at different temperatures (5–18 °C) [16,32]. The WT calculated for muscle tissue varies from 10 to 230 days in other fish species [16]. Fish are poikilothermic animals, and the absorption, distribution, metabolism, and excretion of drugs are temperature-dependent [32]. The WT duration of OTC in plasma and tissues was shortened due to the increase in water temperature [32]. Additionally, the difference in WT in rainbow trout may be due to differences in body size and dosage regimen, as well as temperature.

Pharmacokinetic/pharmacodynamic (PK/PD) modeling is used to determine the optimal dosage regimen of antibacterial drugs. PK/PD data such as AUC/minimum inhibitory concentration (MIC), C_max_/MIC, and T > MIC are used to evaluate the clinical efficacy of OTC [16,45]. However, there is no information on what these values should be to achieve the desired effect. Additionally, it is difficult to determine appropriate PK/PD values for tetracyclines because their in vitro and in vivo MIC values differ and their binding to plasma proteins is atypical and nonlinear [45]. Therefore, we evaluated the data of OTC concentration over the MIC, which is a classical approach. The MIC of OTC for susceptible bacteria from different fish species has been reported to vary between 0.125 and 0.78 µg/mL [16] and the susceptible breakpoint of OTC is ≤1 µg/mL [46]. After single oral administration of OTC to rainbow trout at a dose of 60 mg/kg, the plasma concentration was above the susceptible breakpoint value from 4 h to 72 h. Considering these data, the dosing interval may be longer than 24 h in OTC therapy, of which more than 90% is excreted unchanged in the feces, to reduce therapeutic costs and environmental pollution. However, more detailed PK/PD studies are needed on this subject.

One of the limitations of this study is that oral administration was performed by gastric gavage. We chose this route of administration to determine the single-dose and multiple-dose pharmacokinetics of OTC in rainbow trout. However, in WT studies, it is recommended to perform the administration of OTC as medical feed. In medical feed administrations, reasons such as reactivity with the aquatic environment, leakage from pellets, poor palatability of medicated food, and floating time of pellets may change the pharmacokinetics of drugs. For this reason, WTs obtained after gastric gavage and medical feed administration may differ.

## 5. Conclusions

The single oral administration of OTC exhibited a long half-life and very low bioavailability. In multiple-dose treatment, t_1/2ʎz_, AUC_0–∞_, and C_max_ increased significantly after the last day compared to the first day and OTC showed strong accumulation. At 9 ± 0.8 °C, the WT for safe muscle+skin consumption should not be less than 56 days in the EU and 50 days in China. The results of this study suggest that in OTC multiple-dose treatment, the dosing interval may be longer than 24 h to ensure positive effects such as reducing treatment costs and environmental pollution. However, further studies are required to determine the optimal dosage regimen in OTC multiple-dose treatment and to obtain suitable PK/PD data for the treatment of infections caused by susceptible pathogens.

## Figures and Tables

**Table 1:** Plasma pharmacokinetic parameters of oxytetracycline following single intravenous (IV) and oral administrations of 60 mg/kg dose in rainbow trout at 9 ± 0.8 °C (n = 6).

Parameters	IV	Oral	%GD *
t_1/2ʎz_ (h)	65.05	68.94	5.98
AUC_0–last_ (h·µg/mL)	7309.79	203.53	−97.22
AUC_0–∞_ (h·µg/mL)	7421.10	208.11	−97.20
AUC_extrap_ (%)	1.50	2.20	-
MRT_0-∞_ (h)	74.61	105.62	41.56
Cl_T_ (mL/h/kg)	8.09	-	-
V_dss_ (mL/kg)	603.23	-	-
V_darea_ (mL/kg)	758.74	-	-
C_0.25h_ (µg/mL)	825.39 ± 96.75	-	-
C_max_ (µg/mL)	-	1.61 ± 0.25	-
T_max_ (h)	-	12	
F (%)	-	2.80	-

* refers to percentage (%) difference of oral administration compared to IV administration [100 × (Oral − IV)/IV]. t_1/2ʎz_, elimination half-life; AUC, area under the concentration–time curve; AUC_extrap_ %, area under the plasma concentration–time curve extrapolated from tlast to ∞ in % of the total AUC; MRT, mean residence time; Cl_T_, total clearance; V_dss_, volume of distribution at steady state; V_darea_, apparent volume of distribution; C_0.25h_, plasma concentration at time 0.25 h; C_max_, peak plasma concentration; T_max_, time to reach the peak plasma concentration; F, absolute bioavailability.

**Table 2:** Plasma pharmacokinetic parameters of oxytetracycline following multiple-dose (every 24 h for 7 days) oral administration of 60 mg/kg in rainbow trout at 9 ± 0.8 °C (n = 6).

Parameters	Oral
t_1/2ʎz_ (h)	147.26
AUC_0–1080_ (h·µg/mL)	1978.98
AUC_0–∞_ (h·µg/mL)	1986.02
AUC_extrap_ (%)	0.35
MRT_0–∞_ (h)	230.20
C_max_ (µg/mL)	7.82 ± 0.42
T_max_ (h)	12

t_1/2ʎz_, elimination half-life; AUC, area under the concentration–time curve; AUC_extrap_ %, area under the plasma concentration–time curve extrapolated from tlast to ∞ in % of the total AUC; MRT, mean residence time; C_max_, peak plasma concentration; T_max_, time to reach the peak plasma concentration.

**Table 3:** Plasma pharmacokinetic parameters on the first (1 dose) and last day (7 doses) of oxytetracycline following multiple-dose oral administration.

Parameters	First Dose	Last Dose	%GD *
AUC_0–24_ (h·µg/mL)	32.71	174.38	433.06
C_max_ (µg/mL)	1.60 ± 0.18	7.82 ± 0.42	390.15
C_24h_ (µg/mL)	1.46 ± 0.16	7.28 ± 0.43	397.53
R	-	5.33	

* refers to percentage (%) difference of last-day administration compared to first-day administration [100 × (last dose − first dose)/first dose]. AUC, area under the concentration–time curve; C_max_, peak plasma concentration; C_24h_, plasma concentration at time 24 h; R, accumulation ratio.

**Table 4:** Plasma and tissue concentrations of oxytetracycline in rainbow trout (*Oncorhynchus mykiss*) following multiple-dose (every 24 h for 7 days) oral administration of 60 mg/kg in rainbow trout at 9 ± 0.8 °C (n = 6, mean ± SD).

Time (hour)	Muscle+Skin (µg/g)	Liver (µg/g)	Kidney (µg/g)	Plasma (µg/mL)
0.25	10.65 ± 1.23	87.33 ± 12.98	12.82 ± 1.44	5.50 ± 0.48
0.5	12.52 ± 1.31	105.40 ± 11.96	14.55 ± 1.49	5.86 ± 0.46
1	14.03 ± 1.58	135.77 ± 11.22	16.52 ± 1.10	6.18 ± 0.44
2	15.59 ± 1.60	151.19 ± 16.50	18.70 ± 1.16	6.56 ± 0.39
4	17.14 ± 1.71	172.66 ± 23.41	21.00 ± 1.41	6.91 ± 0.37
8	18.23 ± 1.41	162.36 ± 15.19	24.40 ± 1.59	7.34 ± 0.34
12	17.43 ± 0.78	152.96 ± 12.84	23.20 ± 1.61	7.82 ± 0.42
24	15.83 ± 0.61	128.54 ± 12.96	21.45 ± 1.97	7.28 ± 0.43
48	13.81 ± 0.68	108.39 ± 10.15	19.16 ± 1.81	6.79 ± 0.46
72	12.42 ± 0.68	87.92 ± 9.65	17.17 ± 1.95	6.26 ± 0.51
96	10.94 ± 0.78	67.36 ± 8.43	15.13 ± 1.79	5.69 ± 0.49
120	9.38 ± 0.75	54.68 ± 8.67	13.20 ± 1.68	5.12 ± 0.46
144	7.96 ± 0.69	41.37 ± 4.99	11.30 ± 1.23	4.55 ± 0.45
192	6.30 ± 0.60	33.18 ± 3.40	9.45 ± 0.95	3.83 ± 0.34
240	4.75 ± 0.56	25.13 ± 2.91	7.62 ± 0.71	3.05 ± 0.33
360	3.25 ± 0.43	17.84 ± 2.03	6.09 ± 0.67	1.98 ± 0.22
480	2.40 ± 0.25	11.77 ± 0.59	4.52 ± 0.48	1.23 ± 0.16
600	1.73 ± 0.13	6.33 ± 0.60	3.26 ± 0.39	0.69 ± 0.12
720	1.13 ± 0.12	3.68 ± 0.51	2.21 ± 0.35	0.42 ± 0.11
840	0.63 ± 0.08	2.24 ± 0.26	1.45 ± 0.26	0.21 ± 0.05
960	0.32 ± 0.06	1.27 ± 0.17	0.91 ± 0.13	0.10 ± 0.03
1080	0.15 ± 0.03	0.79 ± 0.13	0.49 ± 0.07	0.04 ± 0.01
1200	0.04 ± 0.02	0.37 ± 0.06	0.23 ± 0.04	<LLOQ
1440	<LLOQ	0.12 ± 0.02	0.09 ± 0.03	<LLOQ
1920	<LLOQ	<LLOQ	<LLOQ	<LLOQ

<LLOQ, below lower limit of quantification of 0.04 μg/mL or μg/g.

**Table 5:** Calculated withdrawal times of oxytetracycline following multiple-dose (every 24 h for 7 days) oral administration of 60 mg/kg in rainbow trout at 9 ± 0.8 °C (n = 6).

Tissues	Europe	China
Muscle+skin	55.87 (56)	49.32 (50)

## Data Availability

The data presented in this study are available on request from the corresponding author.
